# A Novel Microfluidic Assay for Rapid Phenotypic Antibiotic Susceptibility Testing of Bacteria Detected in Clinical Blood Cultures

**DOI:** 10.1371/journal.pone.0167356

**Published:** 2016-12-14

**Authors:** Christer Malmberg, Pikkei Yuen, Johanna Spaak, Otto Cars, Thomas Tängdén, Pernilla Lagerbäck

**Affiliations:** 1 Department of Medical Sciences, Section of Infectious Diseases, Uppsala University, Uppsala, Sweden; 2 Gradientech AB, Uppsala, Sweden; University of South Australia, AUSTRALIA

## Abstract

**Background:**

Appropriate antibiotic therapy is critical in the management of severe sepsis and septic shock to reduce mortality, morbidity and health costs. New methods for rapid antibiotic susceptibility testing are needed because of increasing resistance rates to standard treatment.

**Aims:**

The purpose of this study was to evaluate the performance of a novel microfluidic method and the potential to directly apply this method on positive blood cultures.

**Methods:**

Minimum inhibitory concentrations (MICs) of ciprofloxacin, ceftazidime, tigecycline and/or vancomycin for *Escherichia coli*, *Pseudomonas aeruginosa*, *Klebsiella pneumoniae* and *Staphylococcus aureus* were determined using a linear antibiotic concentration gradient in a microfluidic assay. Bacterial growth along the antibiotic gradient was monitored using automated time-lapse photomicrography and growth inhibition was quantified by measuring greyscale intensity changes in the images. In addition to pure culture MICs, vancomycin MICs were determined for *S*. *aureus* from spiked and clinical blood cultures following a short centrifugation step. The MICs were compared with those obtained with the Etest and for *S*. *aureus* and vancomycin also with macrodilution.

**Results:**

The MICs obtained with the microfluidic assay showed good agreement internally as well as with the Etest and macrodilution assays, although some minor differences were noted between the methods. The time to possible readout was within the range of 2 to 5 h.

**Conclusions:**

The examined microfluidic assay has the potential to provide rapid and accurate MICs using samples from positive clinical blood cultures and will now be tested using other bacterial species and antibiotics.

## Introduction

Severe sepsis and septic shock are life-threatening conditions associated with high morbidity and mortality [1]. It has been shown that delayed appropriate antibiotic therapy is strongly associated with higher mortality, prolonged hospitalisation and higher costs [2–3]. The increasing prevalence of bacteria resistant to standard empirical treatment coupled with the lack of rapid susceptibility testing poses a major clinical challenge [4]. New rapid diagnostic tools are urgently needed to provide real-time information on antibiotic susceptibility that can be used to tailor antibiotic therapy to cover resistant strains and de-escalate therapy in a timely manner to avoid excessive treatment with broad-spectrum antibiotics, side effects and emergence of resistance [1].

Standard culture-based methods for the detection of bacteria, species determination and antibiotic susceptibility testing (AST) are sensitive but too slow to guide the choice of empirical therapy. Much of the current research in new AST methods is focused on genotypic characterisation of antibiotic-resistance (e.g., genetic probes, microarrays, nucleic acid amplification techniques and DNA sequencing) [5]. These techniques provide a shortened time to detection of specific known resistance genes. However, genotypic tests are inherently limited by the need for *a priori* knowledge about the target resistance genes, as well as their inability to quantify gene expression levels and identify the combined effects of multiple resistance mechanisms [5–6]. In contrast, phenotypic methods determine the bacterial susceptibility to the tested antibiotics, but do not provide any information on the mechanisms of resistance present in the causative pathogen. Furthermore, the time to readout is typically longer with phenotypic methods because they depend on bacterial growth to determine the effects of the tested antibiotics. Another weakness with traditional phenotypic methods like Etest and macrodilution is a poor resolving power between wildtype and heteroresistant strains with resistant subpopulations such as heteroresistant vancomycin-intermediate *S*. *aureus* (hVISA). Confirmatory testing with population analysis remains the golden standard for these strains but the experimental procedure is time consuming, labor intensive and unsuitable for routine use in the clinical laboratory setting.

Here, we have evaluated the CellDirector® 3D assay (Gradientech AB, Uppsala, Sweden) as a method for the rapid AST of bacteria. CellDirector 3D is a cell-based assay using microfluidics to generate a linear gradient of substances by diffusion through a microscale test chamber [7]. The principle of this assay is analogous to contemporary phenotypic methods such as the Etest by which bacterial growth in the presence of an antibiotic gradient is detected. In a previous study the CellDirector 3D assay was shown to accurately measure the MICs for *Escherichia coli*, *Staphylococcus aureus* and *Salmonella* Typhimurium of five antibiotics within 4 h and to quantify antibacterial pharmacodynamics with very high precision for *S*. Typhimurium exposed to streptomycin [7].

In the present study the lower detection threshold and optimal bacterial concentration to accurately measure the MIC in the CellDirector 3D were determined using *S*. *aureus* with vancomycin and *Pseudomonas aeruginosa* with ciprofloxacin. Further tests were made using quality control strains of *E*. *coli*, *Klebsiella pneumoniae* and *S*. *aureus* exposed to ciprofloxacin, ceftazidime, tigecycline and/or vancomycin. Finally, the assay was evaluated for applicability directly on positive *S*. *aureus* blood cultures.

## Materials and Methods

### Bacterial strains

Quality control strains of *Pseudomonas aeruginosa* (ATCC 27853), *Staphylococcus aureus* (vancomycin susceptible *S*. *aureus* [VSSA] ATCC 29213 and heteroresistant vancomycin-intermediate *S*. *aureus* [hVISA] ATCC 700698, defined as MIC ≤2 mg/L but with a subpopulation with MIC >2 mg/L), *Escherichia coli* (ATCC 25922) and *Klebsiella pneumoniae* (ATCC 29665) were used for initial evaluation of the CellDirector 3D assay. All strains were stored at –80°C and streaked on Mueller-Hinton II agar plates (MHII, Becton Dickinson, Oxford, UK) (*E*. *coli*, *P*. *aeruginosa*, *K*. *pneumoniae*) or blood agar plates (*S*. *aureus*) before the experiments.

Aliquots from BacT/Alert® FA Plus aerobic blood culture bottles (bioMérieux, Marcy l’Etoile, France) containing 30 mL growth medium and 5–10 mL blood from suspected sepsis patients were provided after incubation and upon bacterial detection in the BacT/Alert® 3D automated microbial detection system (bioMérieux, Marcy l’Etoile, France). The aliquots were kept at room temperature until *S*. *aureus* typing had been confirmed by coagulase test and microscopy (typically under 30 min), and then stored at 4°C for up to 4h. In all, 13 positive blood cultures containing *S*. *aureus* collected consecutively over a period of 4 weeks (May 2014) at the Department of Clinical Microbiology, Uppsala University Hospital were used in the evaluation of applicability directly on positive blood cultures.

### Antibiotics

Stock solutions (10,000 mg/L) of ceftazidime and vancomycin were made in phosphate saline buffer (PBS) while ciprofloxacin was dissolved in 0.1M HCl and tigecyclinein dimethyl sulfoxide (DMSO). All antibiotics were from Sigma-Aldrich, MO, USA and stock solutions were kept at 4°C for a maximum of 4 days, except for tigecycline, which was stored at -20°C. The stock solutions were diluted in PBS to achieve 10x the desired concentration and then further diluted at a 1:10 ratio in MHII broth before injection into the CellDirector 3D assay. Freshly prepared MHII broth was always used with tigecycline. The antibiotic concentration intervals tested in CellDirector 3D are listed in Table 1.

**Table 1:** Antibiotic concentration ranges used in CellDirector 3D.

Strain		Antibiotic	Concentration range (mg/L)
*E*. *coli*		ciprofloxacin	0–0.025
		ceftazidime	0–0.05
		tigecycline	0–0.2
*P*. *aeruginosa*		ciprofloxacin	0–0.5
		ceftazidime	0–2
*K*. *pneumoniae*		ciprofloxacin	0–0.016
*S*. *aureus*		vancomycin	0–4*
	spiked blood samples	vancomycin	0–4*
	clinical blood samples	vancomycin	0–4

* In some experiments a range up to 8 mg/L was used.

### Preparation of pure culture samples

Bacterial inocula were prepared by picking 2–5 colonies from an agar plate and adjusting to a 0.5 McFarland standard in MHII broth. For the quantification of the lower detection threshold and optimal bacterial concentration, *S*. *aureus* (VSSA) and *P*. *aeruginosa* suspensions were diluted 5x, 50x, 500x or 5000x in MHII to obtain bacterial concentrations of 10^6^ to 10^3^ CFU/mL in CellDirector 3D. These concentrations of 10^6^ to 10^3^ CFU/mL were then tested against vancomycin (*S*. *aureus*) and ciprofloxacin (*P*. *aeruginosa*). For the remaining tests based on quality control strains in CellDirector 3D (*P*. *aeruginosa* with ceftazidime, *K*. *pneumoniae* with ciprofloxacin, *E*. *coli* with ceftazidime, ciprofloxacin and tigecycline and *S*. *aureus* (VSSA and hVISA) with vancomycin), the bacterial suspension was diluted 50 times in MHII broth and mixed 1:1 with 0.5% w/v Top Vision low melting point agarose (Thermo Fisher Scientific, MA, USA) to obtain a final inoculum of approximately 10^5^ CFU/mL in CellDirector 3D. All experiments were done in triplicate.

### Extraction of bacteria from spiked blood bottles

Colonies of *S*. *aureus* (VSSA and hVISA) were suspended in PBS to 1.0 McFarland standard. 400 μl of the suspension were mixed with 10 mL defibrinated horse blood (sourced locally) and then added to a BacT/Alert® FA Plus aerobic blood culture bottle (Biomérieux, Craponne, France) containing 30 mL growth medium. After 30 min of incubation at 37°C, 2 mL of the culture was centrifuged for 5 min at room temperature at 170 RCF (relative centrifugal force, Universal 320 Benchtop Centrifuge, rotor 1324, Hettich Lab Technology, Tuttlingen, Germany) to separate bacteria from blood cells. A 100 μL sample was plated on MHII agar before and after centrifugation for colony counting to estimate the loss of bacteria. The supernatant was mixed 1:1 with 0.5% w/v Top Vision low melting point agarose and used in CellDirector 3D. All experiments were done in triplicate.

### Extraction of bacteria from clinical blood bottles

Upon indication of growth of *S*. *aureus*, 2 mL samples from clinical blood bottles were provided by the Department of Clinical Microbiology at Uppsala University Hospital. Each sample was streaked on blood agar and 100 μL were plated on MHII agar for colony counting. The remaining sample was centrifuged for 5 min at 170 RCF at room temperature and 100 μL of the supernatant were plated on MHII agar to estimate bacterial loss. The supernatant was diluted 1:10 in MHII and mixed 1:1 with 0.5% w/v Top Vision low melting point agarose and tested once in CellDirector 3D. Single colonies from the blood plate were used to create a frozen stock and for MIC testing with the Etest and macrodilution methods.

### CellDirector 3D assay

**Fig 1:**
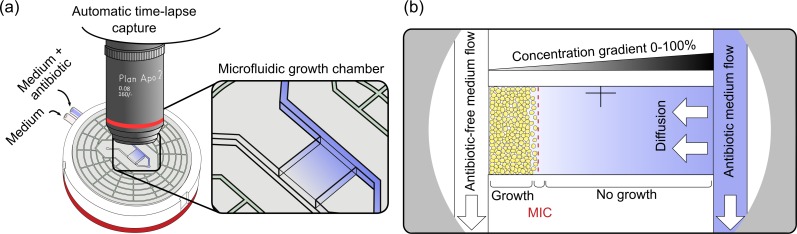
The CellDirector 3D assay. a) Schematic overview of the CellDirector 3D assay setup for AST determination and b) close-up of the microfluidic growth chamber depicting details of the concentration gradient and MIC readout.

The complete CellDirector 3D blister package, including a microfluidics chamber, tubes and syringes was placed in a vacuum chamber for 30 min to counteract formation of microbubbles in the subsequently added agarose matrix. Bacteria/agarose mixtures were prepared as described in previous sections. Using reverse pipetting, 8.5 μl of the bacteria/agarose mixture were injected into the cell culture chamber of the assay (Fig 1) and allowed to solidify for approximately 10 min. The agarose immobilizes the bacteria and prevents growth out into the media channels, as well as provides a medium through which the antibiotic gradient is formed.

A disposable 1 mL syringe was filled with MHII broth while a second syringe was filled with MHII broth containing the maximum concentration of antibiotic to be tested. The antibiotic concentrations in the two syringes determine the minimum and maximum concentrations of the linear gradient established in the assay chamber. An infusion syringe pump (Harvard P-22, Holliston, MA, USA) was used to induce flow through the assay at a pump rate of 2 μl/min. The assay was placed upside down in an upright microscope (Nikon Optiphot-2, Tokyo, Japan) in a 37°C incubator room. Digital images were automatically collected every 10 min for up to 6 h using a Canon EOS D700 DSLR camera with a 2x dark field objective. Images were acquired as 24-bit JPEGs and analysed to determine the MIC as described in the following section. All MICs from CellDirector 3D were compared with those obtained from the Etest and/or macrodilution; a ≤1 log2 deviation from macrodilution was considered acceptable since this is an accepted variation between experiments for both the Etest and macrodilution according to CLSI.

### Image analysis

**Fig 2:**
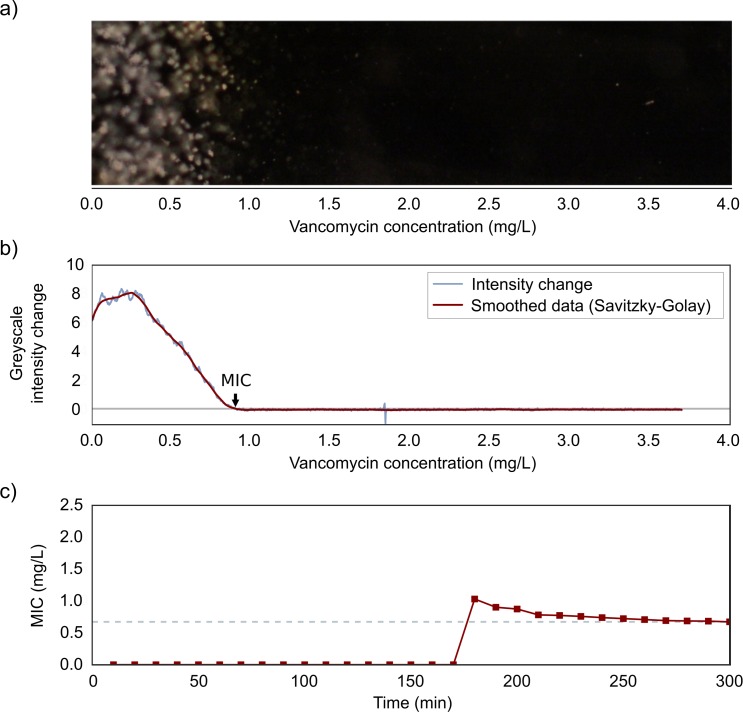
Growth rate analysis and MIC determination from CellDirector 3D images. Example of (a) a dark field image of the cell culture chamber after 300 min in the CellDirector 3D assay and (b) the corresponding plot of greyscale intensity change from the five images over the last 40 min (i.e. growth rate) for *S*. *aureus* (VSSA) and vancomycin. The vancomycin concentration on the x-axis of the plot corresponds to a pixel coordinate on the image x-axis. The MIC value (arrow) is set as the first position at which the change in greyscale intensity is below 0.1 (grey line). In the bottom panel (c) the computed MIC value is plotted for each time point. The MIC is not calculated (and is plotted as 0) until a growth rate signal above 0.1 is detected (at 160 min in the example above) and is stable after this point. The grey dotted line indicates the MIC at 5 h for this strain and antibiotic.

A programme was written to automatically analyse images after CellDirector 3D experiments using Python 2.7.10 (https://www.python.org/), and the packages SciPy 0.16.1, NumPy 1.9.3, pandas 0.17.0, scikit-image 0.11.3 and matplotlib 1.5.0rc3. Images were read and converted to 8-bit greyscale images. For each pixel position along the x-axis (i.e. along the linear antibiotic concentration gradient), the mean intensity level was computed for all pixels in the y-axis of that x-position (Fig 2). The mean intensity level of the initial frame (t = 0) was subtracted from all frames to remove background intensity. The mean greyscale intensity change was computed using the NumPy function gradient, which is based on a second-order central difference. The growth rate at time point *t* was calculated by computing the mean of the greyscale intensity change during the 5 most recent time points (corresponding to 40 min). Assuming a linear gradient, the image pixel coordinates were translated to antibiotic concentrations using the following formula:

$$Antibiotic\;conc.\;at\;pixel\;x=\frac{maximum\;antibiotic\;conc.}{image\;pixel\;width}*pixel\;x\;coordinate$$

A Savitzky-Golay smoothing filter (window size = 151, polyn = 3) was applied and plotted against pixel position (antibiotic concentration gradient). The MIC was determined as the first position at which the growth rate had a value of <0.1.

### MIC determination by Etest

MIC determination by Etest (bioMérieux, Marcy l’Etoile, France) was performed according to the manufacturer’s instructions. 2–5 colonies were suspended in saline and adjusted to a 0.5 (all strains) and 2.0 (hVISA and VISA) McFarland standard and then swabbed on MHII agar plates. A ciprofloxacin, ceftazidime, tigecycline or vancomycin Etest strip was applied on each plate. The MIC was read at the intersection of the inhibition zone and Etest scale after incubation at 37°C for 16–20 h. All strains were tested in at least triplicate on separate occasions, and the modal MIC calculated.

### MIC determination by macrodilution

Macrodilution was performed for vancomycin against all strains of *S*. *aureus* included in the study. A serial dilution of vancomycin ranging from 0.0625 mg/L to 32 mg/L was prepared in MHII broth. The tubes were inoculated with a 0.5 McFarland bacterial suspension to yield a final concentration of ca. 5x10^5^ CFU/mL and incubated overnight at 37°C. MIC was read as the lowest concentration of the drug at which no turbidity could be detected visually. Each *S*. *aureus* isolate was tested in at least triplicate on separate occasions, and the modal MIC calculated.

### Population analysis

Population analysis was performed with the VSSA and hVISA strains. Bacteria were incubated overnight in MHII broth and then diluted to achieve a bacterial concentration of 10^2^−10^8^ CFU/mL. 100 μL of the dilutions were plated onto antibiotic-free plates (control) and plates containing 0.25, 0.5, 1, 2, 3, 4 and 5 mg/L vancomycin. Plates were incubated overnight at 37°C and colonies were counted the next day. The population analysis was done in duplicate. Subpopulation frequencies were calculated by dividing the amount of bacteria growing on plates containing 2 mg/L vancomycin with the amount of bacteria growing on the antibiotic-free plates.

### Statistical analysis

CellDirector 3D MIC values for pure cultures and spiked blood samples of VSSA and hVISA were compared using 2-way ANOVA with Tukeys multiple comparison post test using GraphPad Prism version 6.05 for Windows (GraphPad Software, La Jolla California USA). Results with p < 0.05 were considered statistically significant.

## Results

### Lower detection threshold and inoculum effects in CellDirector 3D

**Fig 3:**
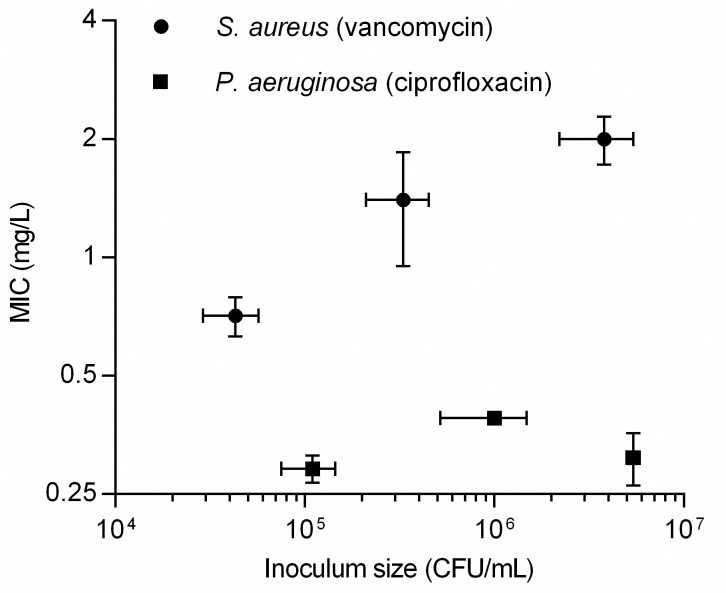
MIC values of VSSA and *P*. *aeruginosa* at different inocula. To determine the lower detection threshold and optimal bacterial concentrations in CellDirector 3D, a range of 10^3^ to 10^6^ CFU/mL for *S*. *aureus* (VSSA) and *P*. *aeruginosa* was tested against vancomycin and ciprofloxacin, respectively (for inocula <10^4^ CFU/mL no MIC could be determined). Circles—VSSA and vancomycin, squares—*P*. *aeruginosa* and ciprofloxacin, error bars signify standard deviation.

**Fig 4:**
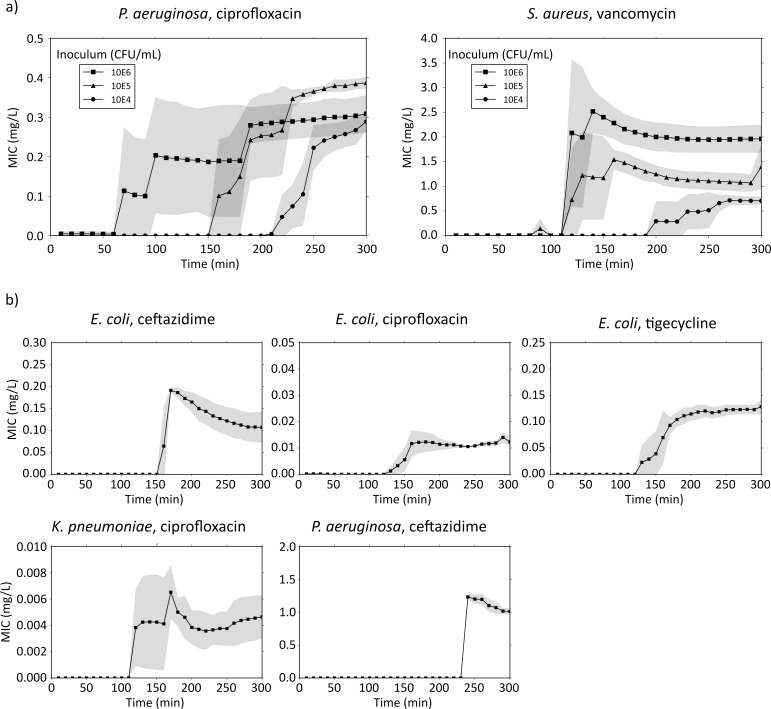
MIC over time for quality control strains. a) MIC determination and stabilisation over time for different inoculum sizes of *S*. *aureus* (VSSA) and *P*. *aeruginosa*, tested against vancomycin and ciprofloxacin, respectively. b) MIC over time for *E*. *coli* against ceftazidime, ciprofloxacin and tigecycline, *K*. *pneumoniae* against ciprofloxacin, and *P*. *aeruginosa* against ceftazidime, at an inoculum size of 10^5^ CFU/mL. Grey fields signify standard deviation (n = 3).

Based on the bacterial density in the CellDirector 3D chamber and the density-related robustness of the data analysis, the optimal inoculum size in CellDirector 3D was determined to be ca. 10^5^ CFU/mL and the detection threshold ca. 10^4^ CFU/mL. A lower inoculum size (<10^4^ CFU/mL) resulted in no or insufficient detection of growth and no MIC value could be determined, whereas a higher inoculum size (10^6^ CFU/mL) resulted in consistently higher MICs due to inoculum effects with vancomycin against *S*. *aureus* (Fig 3). For *S*. *aureus* (VSSA) with vancomycin, the mean MICs measured in CellDirector 3D after 5 h were 0.71 (SD, 0.12), 1.4 (SD, 0.45) and 2.0 (SD, 0.28) mg/L for an inoculum size of ca. 4x10^4^, 4x10^5^ and 4x10^6^ CFU/mL, respectively. For *P*. *aeruginosa* with ciprofloxacin, the mean MICs measured in CellDirector 3D were 0.29 (SD, 0.023), 0.39 (SD, 0.014) and 0.31 (SD, 0.047) mg/L with bacterial inocula of ca. 1x10^5^, 1x10^6^ and 5x10^6^ CFU/mL, respectively. The MICs obtained with the Etests were 1.5 mg/L for *S*. *aureus* (VSSA) with vancomycin and 0.19 mg/L for *P*. *aeruginosa* with ciprofloxacin. The difference in MICs as compared with the corresponding CellDirector 3D values were within 1 log2, except for *S*. *aureus* at 4 x10^4^ CFU/mL, where the difference was slightly greater (0.71 mg/L for the CellDirector 3D MIC method vs. 1.5 for the Etest) and for *P*. *aeruginosa* with ciprofloxacin at 1x10^6^ CFU/mL (0.39 mg/L for the CellDirector 3D MIC method vs. 0.19 for the Etest). For VSSA, the macrodilution MIC of vancomycin was 1.0 mg/L, which was within 1 log2 difference from all CellDirector 3D MICs. The presented MICs were determined after 5 h in the CellDirector 3D assay, but in some cases similar MICs (defined as <15% variation from end result) could have been read already after 2–5 h (Fig 4A, S1 Table).

### Validation using quality control strains

Based on the results of the optimal inoculum size, further experiments were made using an inoculum size of 10^5^ CFU/mL to validate the performance of the CellDirector 3D method with other strains and antibiotics (Table 2, S4 Table). All CellDirector 3D MICs were within 1 log2 difference from the corresponding Etest MIC. For these strains and antibiotics, the potential time to readout varied between 3–5 h (Fig 4B, S2 Table).

**Table 2:** MIC values in mg/L obtained with the Etest and CellDirector 3D at bacterial inocula of 10^5^ CFU/mL.

Strain	Antibiotic	Etest MIC (range)	CellDirector 3D MIC (SD)
*S*. *aureus* (VSSA)	vancomycin	1.5 (1.5)	1.4 (0.45)
*P*. *aeruginosa*	ciprofloxacin	0.19 (0.125–0.25)	0.39 (0.014)
*P*. *aeruginosa*	ceftazidime	1.0 (0.75–1)	1.0 (0.050)
*E*. *coli*	ciprofloxacin	0.008 (0.006–0.008)	0.013 (0.0014)
*E*. *coli*	ceftazidime	0.19 (0.125–0.19)	0.11 (0.034)
*E*. *coli*	tigecycline	0.19 (0.125–0.19)	0.13 (0.013)
*K*. *pneumoniae*	ciprofloxacin	0.006 (0.004–0.008)	0.0046 (0.0016)

### MIC determination and population analysis using pure cultures of VSSA and hVISA

**Fig 5:**
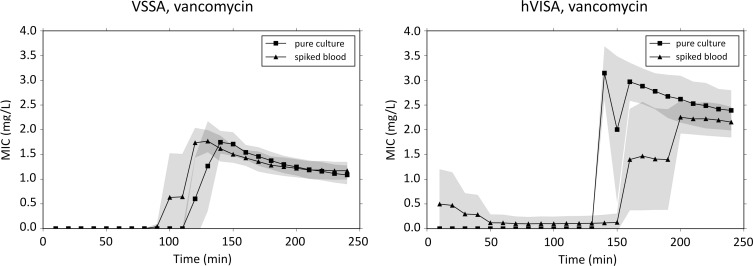
MIC over time for VSSA and hVISA. MIC determination and stabilisation over time for *S*. *aureus* (VSSA and hVISA) from pure culture and spiked blood bottles. Grey fields signify standard deviation (n = 3).

In repeated experiments with the VSSA strain at an inoculum of 2x10^5^ CFU/mL the MICs against vancomycin with the CellDirector 3D (determined after 4 h), Etest and macrodilution were 1.1 (SD, 0.19), 1.5 and 1.0 mg/L, respectively (Table 3, S5 Table). Vancomycin MICs for hVISA were 2.4 (SD, 0.41) mg/L with the CellDirector 3D and 2.0 mg/L with the Etest and macrodilution broth method. An increase in bacterial inoculum size from 0.5 to 2 McFarland in an attempt to capture the hVISA subpopulation with the Etest did not change the results. The potential time to results in the CellDirector 3D ranged from 3–4 h (Fig 5, S3 Table). In the population analysis the detected subpopulation frequencies of the VSSA and hVISA strains growing on agar plates containing 2 mg/L of vancomycin were 5x10^-8^ and 6x10^-5^, respectively (S1 Fig).

**Table 3:** Vancomycin MIC values in mg/L as determined by the Etest, macrodilution and CellDirector 3D against VSSA and hVISA.

Strain		Etest (range)	Macrodilution (range)	CellDirector 3D (SD)
VSSA				
	pure culture	1.5 (1.5)	1.0 (0.5–1)	1.1 (0.19)
	spiked blood			1.2 (0.18)
hVISA				
	pure culture	2.0 (2)	2.0 (1–2)	2.4 (0.41)
	spiked blood			2.2 (0.31)

### MIC determination of VSSA and hVISA from spiked blood bottles

The centrifugation step used for spiked blood bottle samples to separate the bacteria from the blood resulted on average in 43% (n = 6; SD, 20%; S6 Table) recovery of bacteria corresponding to an average bacterial concentration of 2x10^6^ CFU/mL in the supernatant and 1x10^6^ CFU/mL in the CellDirector 3D chamber. The MICs for VSSA and hVISA from spiked blood bottles were 1.2 (SD, 0.18) and 2.2 (SD, 0.31) mg/L, respectively, after 4 h in CellDirector 3D (Table 3). Some erythrocytes and cell debris remained after the centrifugation step but did not have any major effect on the result, compared to the MICs obtained from pure cultures there was <0.2 mg/L difference between the mean values (Table 3), and the difference was not statistically significant (VSSA: p > 0.99, hVISA: p = 0.97).

### MIC determination from clinical blood cultures containing *S*. *aureus*

The supernatant from the 13 clinical blood cultures contained *S*. *aureus* at concentrations ranging from 3x10^6^ to 2x10^8^ CFU/mL as determined by viable counts. The average loss of bacteria after centrifugation was in most cases less than 1 log2 CFU/mL (28% recovery; n = 13; SD, 33%). The concentration in the assay chamber was thus ca. 1x10^5^ to 9x10^6^ CFU/mL after dilution (Table 4). In these experiments the MIC was read at 4 h and the MIC results for 9 of the 13 strains in CellDirector 3D were within 1 log2 deviation as compared with the Etest, and 10 of 13 were within 1 log2 deviation as compared with the macrodilution method (Table 4). For lower CellDirector 3D inocula (<ca. 5x10^5^ CFU/mL), there was a trend towards lower MIC values compared with macrodilution, and for higher inocula a trend towards higher MIC values. The Etest MICs were higher than those of the macrodilution assay in 9 of 13 cases. The total time to readout upon collection of positive *S*. *aureus* blood cultures for the CellDirector 3D assay was typically within 5 h (S2 Fig), including the centrifugation step (10 min), assay setup (20 min) and analysis of data (3 min).

**Table 4:** Vancomycin MIC values (mg/L) for 13 *S*. *aureus* isolates from clinical blood cultures as determined by the Etest, macrodilution and CellDirector 3D (4 h readout).

Clinical isolate	Etest	Macrodilution	CellDirector 3D	CellDirector 3Dinoculum, CFU/mL
1	1.5	1.0	2.1	5x10^6^
2	2.0	2.0	2.1	2x10^6^
3	2.0	2.0	2.3	9x10^6^
4	1.5	1.0	1.2	8x10^5^
5	2.0	1.0	0.80	4x10^5^
6	1.5	2.0	0.56	2x10^5^
7	2.0	1.0	2.0	4x10^6^
8	1.5	1.0	1.4	3x10^6^
9	2.0	1.0	0.93	7x10^5^
10	2.0	1.0	0.75*	1x10^5^
11	1.5	1.0	1.5	9x10^5^
12	2.0	2.0	2.0	3x10^6^
13	2.0	1.0	2.1	6x10^5^

* Read at 6 h due to no visible growth at 4 h

## Discussion

In the present study we have shown the potential of the CellDirector 3D assay to rapidly determine antibiotic susceptibilities of pathogens frequently detected in clinical blood cultures. We have also shown that it is possible to use samples directly from blood culture bottles in the CellDirector 3D to further decrease the time until a susceptibility result can be obtained.

The detection threshold in the CellDirector 3D was determined by using different inoculum sizes of *S*. *aureus* (against vancomycin) and *P*. *aeruginosa* (against ciprofloxacin). The lower detection threshold was 10^4^ CFU/mL in these experiments and the optimal bacterial concentration approximately 10^5^ CFU/mL. At suboptimal inocula, it takes longer time to provide a stable MIC comparable to the standard methods, if at all possible, and at higher inocula the inoculum effect increased the apparent MIC value (Figs 3 and 4A). The effect of vancomycin against *S*. *aureus* has also previously been shown to be dependent on the inoculum size [8–9]. Because the concentration of bacteria present in the blood stream is usually very low in septic patients (1–100 CFU/mL [10]), prior blood culturing is still necessary to yield a sufficient number of bacteria before MIC determination can be performed. MIC values determined in the CellDirector 3D were within 1 log2 deviation from those of the standard methods (Etest or macrodilution), with the exception of vancomycin against *S*. *aureus* at an inoculum size of 10^4^ CFU/mL and for *P*. *aeruginosa* with ciprofloxacin at 1x10^6^ CFU/mL where the difference was borderline. Since a 1 log2 difference between experiments is considered acceptable for both the Etest and macrodilution, we found this to be an acceptable difference also between methods. Macrodilution is preferred over the Etest with *S*. *aureus* and vancomycin because of the tendency of the Etest to produce higher MIC values as compared with the CLSI standard method of broth dilution [11]. Therefore, macrodilution was performed in addition to the Etest to determine the vancomycin MICs for all *S*. *aureus* strains in this study.

The performance of the CellDirector 3D was also validated using the optimal inoculum size of 10^5^ CFU/mL with *P*. *aeruginosa*, *E*. *coli* and *K*. *pneumoniae* with ceftazidime, ciprofloxacin and/or tigecycline. The MIC values obtained with the microfluidic assay were within 1 log2 deviation from those of the Etest (Table 2). In these experiments MIC results could be obtained within 3–5 h (Fig 4B, S2 Table), which is significantly shorter than the 16–20 h needed for the Etest and macrodilution methods.

The ability to discriminate hVISA from VSSA may be clinically relevant because hVISA has been associated with a higher risk for treatment failure in patients treated with vancomycin or other glycopeptides [12]. However, hVISA is not easily detected with standard methods because of the slow growth and low ratio of the resistant subpopulation and is therefore often unnoticed in clinical practice [13]. Methodological challenges exist with high molecular weight antimicrobials (such as vancomycin) and disc diffusion tests are discouraged because low diffusion rates can make interpretation of results more difficult [14]. Based on one-dimensional diffusion laws and empirical measurements, the time required to establish a linear vancomycin gradient in the CellDirector 3D assay is ~180 min (S1 Text). In our experiments the hVISA subpopulations were present at a frequency of 6x10^-5^ according to the population analysis, which is consistent with the frequency reported in other studies [15–16]. As often is the case, hVISAs were not detected with the Etest or macrodilution methods, even with a higher inoculum of 2 McFarland. In contrast, in the CellDirector 3D assay the hVISA MICs were determined to be >2 mg/L (Table 3). With a total start inoculum of ca. 4x10^3^ CFU in the CellDirector 3D chamber, the heteroresistant subpopulation should theoretically not be detectable. It is nevertheless possible that small, slow growing colonies could be resolved in the microscope but not by the naked eye after 24 h in the population analysis.

Sampling directly from blood culture bottles was feasible with a short centrifugation step. Blood components were still present after centrifugation, but because the analysis is based on growth rate differences and the background debris is subtracted from each frame, non-growing pre-existing debris is likely to have a limited impact on the read-out of the MIC value. MICs determined from spiked blood samples were essentially identical to MICs determined from pure cultures of VSSA and hVISA and with similar standard deviations (Fig 5, Table 3). For the clinical *S*. *aureus* blood isolates, the Etest MICs were frequently higher than the macrodilution MICs, which is in agreement with previous reports [11]. The CellDirector 3D MICs were closer to the Etest MICs than the macrodilution MICs in 6 of 13 cases and were equally close in 3 cases in which the same MIC values were obtained with the Etest and macrodilution methods. The CellDirector 3D results for the clinical blood samples were in most cases within 1 log2 from the Etest MIC (9 of 13 cases) and macrodilution MIC (10 of 13 cases). These inocula were not standardised and there was an apparent inoculum effect in the CellDirector 3D also here (Table 4), using a standardised inoculum would most likely improve the accuracy.

A major advantage of the CellDirector 3D method is that it is significantly faster than the standard phenotypic methods. With improved detection algorithms and microscope resolution, the time to readout could, depending on what bacterial species and antibiotics are used, be reduced to 2–3 h according to preliminary data. The time to readout with this assay depends on the time required to establish a stable antibiotic gradient and the lag time for a measurable antibiotic response in terms of reduced bacterial growth. Of these two limiting factors the first is likely to have the greatest effect in this system. This effect can be seen in Fig 2C as a gradual decrease in MIC until a stable value is reached, which most likely represents the edge of diffusion of the vancomycin MIC concentration in the agarose until the concentration gradient reaches linearity. The time point is also consistent with the calculation of the diffusion rate (S1 Text). In contrast, an effect from antibiotic lag time would show as a more rapid step down in MIC readout after the lag time.

One of the limitations of CellDirector 3D is the relatively narrow concentration range, which is an inherent problem with using a linear assay if a high resolution is to be maintained. However, appropriate selection of antibiotic concentrations centred on the susceptibility breakpoints, would provide clinically relevant results and allow more precise MIC determination than methods based on exponential scales. With traditional methods, the results are typically visually read in two-fold steps and a minor error in the interpretation of bacterial growth may thus have a large impact on the MIC value reported to treating clinicians. In the current format of CellDirector 3D only one isolate can be tested against one antibiotic in each assay and the protocol is relatively labour-intensive. To resolve many of the practical limitations a multi-channel version of the assay is under development.

In conclusion, our results suggest that the CellDirector 3D assay can be used to determine the MICs of the tested antibiotics and species within 2–6 h. The results were highly reproducible and in most cases in agreement with MICs obtained with standard methods (Etest and macrodilution). Following a single centrifugation step, MIC values can be determined directly from blood cultures as was shown with vancomycin against *S*. *aureus* in this study. Further experiments that include other antibiotics and bacterial species using a multichannel assay and improved automatic data analysis are underway.

## Supporting Information

S1 Table MIC values for different inocula of VSSA and *P*. *aeruginosa* at different time points. MIC values (mg/L) as determined from the CellDirector 3D assay at 5, 4, 3, 2 and 1 h and percent agreement compared with the MIC determined at 5 h for (a) *S*. *aureus* with vancomycin and (b) *P*. *aeruginosa* with ciprofloxacin at different inocula.
(PDF)

S2 Table MIC values of quality control strains at different time points. MIC values (mg/L) as determined from the CellDirector 3D assay at 5, 4, 3, 2 and 1 h and percent agreement compared with the MIC determined at 5 h for *P*. *aeruginosa* with ceftazidime, *E*. *coli* with ciprofloxacin, ceftazidime and tigecycline and *K*. *pneumoniae* with ciprofloxacin.
(PDF)

S3 Table MIC values for VSSA and hVISA at different time points. MIC values (mg/L) as determined from the CellDirector 3D assay at 5, 4, 3, 2 and 1h and percent agreement compared to the MIC determined at 5h for *S*. *aureus* VSSA and hVISA (from pure cultures and spiked blood cultures) with vancomycin.
(PDF)

S4 Table MIC values from CellDirector 3D and Etest for quality control strains. MIC values for QC strains determined by Etest and CellDirector 3D from pure cultures at the optimal inoculum size.
(PDF)

S5 Table MIC values from CellDirector 3D, Etest and macrodilution for VSSA and hVISA. MIC values for VSSA and hVISA strains determined by CellDirector 3D, Etest and Macrodilution from pure cultures and from spiked blood culture bottles.
(PDF)

S6 Table Recovery of bacteria from spiked blood bottles. Percent recovery and bacterial concentrations before and after centrifugation of spiked blood bottles.
(PDF)

S7 Table Recovery of bacteria from clinical blood bottles. Percent recovery and bacterial concentrations before and after centrifugation of clinical blood bottles.
(PDF)

S1 Fig Population analysis of VSSA and hVISA with vancomycin. Population analysis of VSSA and hVISA strains using vancomycin agar plates with 0, 0.25, 0.5, 1, 2, 3, 4 and 5 mg/L antibiotic concentration and overnight incubation. The number of surviving colonies represent fraction of resistant sub-populations at the tested concentration. Error bars denote standard deviation (n = 2).
(PDF)

S2 Fig Time to readout in CellDirector 3D for clinical blood cultures. Thirteen isolates of *S*. *aureus* were extracted from blood bottles after positive signal for growth in BacT/Alert® 3D and identification using microscopy and coagulase test. The samples were analysed in the CellDirector 3D system by collecting an image every 10 minutes. The calculated MIC values are plotted for each time point.
(PDF)

S1 Text Diffusion of vancomycin in CellDirector3D. (PDF)
