# Supplementary material for: A Novel Microfluidic Assay for Rapid Phenotypic Antibiotic Susceptibility Testing of Bacteria Detected in Clinical Blood Cultures
Source: PLoS One. 2016 Dec 14;11(12):e0167356. doi: 10.1371/journal.pone.0167356 (PMC5156554; doi:10.1371/journal.pone.0167356)
Supplement: S1 Table — MIC values (mg/L) as determined from the CellDirector 3D assay at 5, 4, 3, 2 and 1 h and percent agreement compared with the MIC determined at 5 h for (a) S. aureus with vancomycin and (b) P. aeruginosa with ciprofloxacin at different inocula. (PDF) [file pone.0167356.s001.pdf]

S1 Table. MIC values for different inocula of VSSA and P. aeruginosa at different time points.

MIC values (mg/L) as determined from the CellDirector 3D assay at 5, 4, 3, 2 and 1 h and percent agreement compared with the MIC determined at 5 h for (a) S. aureus with vancomycin and (b) P. aeruginosa with ciprofloxacin at different inocula.

a)

Inocula (CFU/mL)	5 hours	4 hours	%	3 hours	%	2 hours	%	1 hour	%
10^6	2.27	2.26	100%	2.44	107%	3.35	147%	0.00	0%
10^6	2.01	1.97	98%	2.12	105%	2.90	144%	0.00	0%
10^6	1.60	1.61	101%	1.74	109%	0.00	0%	0.00	0%
10^5	1.30	1.37	105%	1.64	126%	2.18	168%	0.00	0%
10^5	1.98	1.06	53%	1.37	69%	0.00	0%	0.00	0%
10^5	0.90	0.95	105%	1.17	130%	0.00	0%	0.00	0%
10^4	0.80	0.84	105%	0.00	0%	0.00	0%	0.00	0%
10^4	0.72	0.62	86%	0.00	0%	0.00	0%	0.00	0%
10^4	0.60	0.00	0%	0.00	0%	0.00	0%	0.00	0%
10^3	0.00	0.00	-	0.00	-	0.00	-	1.69	-
10^3	-	-	-	-	-	-	-	-	-
10^3	-	-	-	-	-	-	-	-	-

b)

Inocula (CFU/mL)	5 hours	4 hours	%	3 hours	%	2 hours	%	1 hour	%
10^6	0.32	0.30	95%	0,00	0%	0	0%	0	0%
10^6	0.36	0.34	94%	0.34	94%	0.34	93%	0	0%
10^6	0.25	0.23	94%	0.23	93%	0.25	101%	0	0%
10^5	0.38	0.35	91%	0.10	27%	0	0%	0	0%
10^5	0.37	0.35	93%	0	0%	0	0%	0	0%
10^5	0.41	0.38	93%	0.35	85%	0	0%	0	0%
10^4	0.28	0.12	43%	0	0%	0	0%	0	0%
10^4	0.26	0.19	73%	0	0%	0	0%	0	0%
10^4	0.32	0	0%	0	0%	0	0%	0	0%
10^3	-	-	-	-	-	-	-	-	-
10^3	-	-	-	-	-	-	-	-	-
10^3	-	-	-	-	-	-	-	-	-
