# Supplementary material for: A Novel Microfluidic Assay for Rapid Phenotypic Antibiotic Susceptibility Testing of Bacteria Detected in Clinical Blood Cultures
Source: PLoS One. 2016 Dec 14;11(12):e0167356. doi: 10.1371/journal.pone.0167356 (PMC5156554; doi:10.1371/journal.pone.0167356)
Supplement: S2 Table — MIC values (mg/L) as determined from the CellDirector 3D assay at 5, 4, 3, 2 and 1 h and percent agreement compared with the MIC determined at 5 h for P. aeruginosa with ceftazidime, E. coli with ciprofloxacin, ceftazidime and tigecycline and K. pneumoniae with ciprofloxacin. (PDF) [file pone.0167356.s002.pdf]

S2 Table. MIC values of quality control strains at different time points.

MIC values (mg/L) as determined from the CellDirector 3D assay at 5, 4, 3, 2 and 1 h and percent agreement compared with the MIC determined at 5 h for *P. aeruginosa* with ceftazidime, *E. coli* with ciprofloxacin, ceftazidime and tigecycline and *K. pneumoniae* with ciprofloxacin.

	5 hours	4 hours	%	3 hours	%	2 hours	%	1 hour	%
P. aeruginosa ceftazidime	0.94	1.2	125%	0	0%	0	0%	0	0%
	1.0	1.2	118%	0	0%	0	0%	0	0%
	1.0	1.3	122%	0	0%	0	0%	0	0%
E. coli ciprofloxacin	0.012	0.010	88%	0.0087	75%	0	0%	0	0%
	0.014	0.011	77%	0.011	76%	0	0%	0	0%
	0.011	0.011	94%	0.017	157%	0	0%	0	0%
E. coli ceftazidime	0.060	0.085	141%	0.19	312%	0	0%	0	0%
	0.12	0.14	113%	0.17	141%	0	0%	0	0%
	0.14	0.16	114%	0.20	143%	0	0%	0	0%
E. coli tigecycline	0.14	0.13	97%	0.12	90%	0	0%	0	0%
	0.14	0.12	85%	0.10	72%	0	0%	0	0%
	0.11	0.11	97%	0.090	81%	0	0%	0	0%
K. pneumoniae ciprofloxacin	0.0025	0.0020	77%	0.0050	199%	0	275%	0	0%
	0.0065	0.0048	74%	0.0037	57%	0.0045	70%	0	0%
	0.0049	0.0045	92%	0.0063	128%	0	0%	0	0%
