# Supplementary material for: A Novel Microfluidic Assay for Rapid Phenotypic Antibiotic Susceptibility Testing of Bacteria Detected in Clinical Blood Cultures
Source: PLoS One. 2016 Dec 14;11(12):e0167356. doi: 10.1371/journal.pone.0167356 (PMC5156554; doi:10.1371/journal.pone.0167356)
Supplement: S3 Table — MIC values (mg/L) as determined from the CellDirector 3D assay at 5, 4, 3, 2 and 1h and percent agreement compared to the MIC determined at 5h for S. aureus VSSA and hVISA (from pure cultures and spiked blood cultures) with vancomycin. (PDF) [file pone.0167356.s003.pdf]

S3 Table. MIC values for VSSA and hVISA at different time points.

MIC values (mg/L) as determined from the CellDirector 3D assay at 5, 4, 3, 2 and 1h and percent agreement compared to the MIC determined at 5h for *S. aureus* VSSA and hVISA (from pure cultures and spiked blood cultures) with vancomycin.

	Sample	4 hours	3 hours	%	2 hours	%	1 hour	%
VSSA	pure	1.3	1.6	120%	0	0%	0	0%
		0.86	1.2	136%	0	0%	0	0%
		1.1	1.4	127%	1.8	169%	0	0%
	blood	1.4	1.5	110%	2.1	156%	0	0%
		0.93	1.1	114%	1.4	149%	0	0%
		1.2	1.3	106%	1.7	139%	0	0%
hVISA	pure	2.9	3.1	109%	0	0%	0	0%
		2.5	2.9	120%	0	0%	0	0%
		1.9	2.3	122%	0	0%	0	0%
	blood	2.5	0	0%	0	0%	0	0%
		2.3	2.4	107%	0	0%	0	0%
		1.74	1.8	104%	0	0%	0	0%
