# Supplementary material for: A Novel Microfluidic Assay for Rapid Phenotypic Antibiotic Susceptibility Testing of Bacteria Detected in Clinical Blood Cultures
Source: PLoS One. 2016 Dec 14;11(12):e0167356. doi: 10.1371/journal.pone.0167356 (PMC5156554; doi:10.1371/journal.pone.0167356)
Supplement: S4 Table — MIC values for QC strains determined by Etest and CellDirector 3D from pure cultures at the optimal inoculum size. (PDF) [file pone.0167356.s004.pdf]

S4 Table. MIC values from CellDirector 3D and Etest for quality control strains.

MIC values for QC strains determined by Etest and CellDirector 3D from pure cultures at the optimal inoculum size.

		Etest	CellDirector 3D		
**Species, antibiotic**	**Replicate**	**MIC (mg/L)**	**MIC (mg/L)**	**Mean MIC (SD) (mg/L)**	**Inoculum (CFU/mL)**
S. aureus (VSSA), vancomycin	1	1.5	1.30	1.4 (0.45)	$5.0 \cdot 10^5$
	2	1.5	1.98		$2.8 \cdot 10^5$
	3	1.5	0.90		$2.2 \cdot 10^5$
P. aeruginosa, ciprofloxacin	1	0.25	0.38	0.39 (0.014)	$1.1 \cdot 10^6$
	2	0.125	0.37		$1.6 \cdot 10^6$
	3	0.19	0.41		$4.2 \cdot 10^5$
	4	0.19			
P. aeruginosa, ceftazidime	1	1	0.94	1.0 (0.050)	$7.2 \cdot 10^5$
	2	1	1.0		$9.0 \cdot 10^5$
	3	0.75	1.0		$8.4 \cdot 10^5$
E. coli, ciprofloxacin	1	0.008	0.012	0.013 (0.0014)	$2.6 \cdot 10^5$
	2	0.006	0.014		$3.3 \cdot 10^5$
	3	0.008	0.011		$4.7 \cdot 10^5$
E. coli, ceftazidime	1	0.19	0.060	0.11 (0.034)	$4.1 \cdot 10^5$
	2	0.125	0.12		$3.2 \cdot 10^5$
	3	0.19	0.14		$3.3 \cdot 10^5$
E. coli, tigecycline	1	0.094	0.14	0.13 (0.013)	$4.6 \cdot 10^5$
	2	0.19	0.14		$3.8 \cdot 10^5$
	3	0.125	0.11		$4.5 \cdot 10^5$
	4	0.19			
K. pneumoniae, ciprofloxacin	1	0.008	0.0025	0.0046 (0.0016)	$4.4 \cdot 10^5$
	2	0.004	0.0065		$4.2 \cdot 10^5$
	3	0.006	0.0049		$4.2 \cdot 10^5$
	4	0.006			
