# Supplementary material for: A Novel Microfluidic Assay for Rapid Phenotypic Antibiotic Susceptibility Testing of Bacteria Detected in Clinical Blood Cultures
Source: PLoS One. 2016 Dec 14;11(12):e0167356. doi: 10.1371/journal.pone.0167356 (PMC5156554; doi:10.1371/journal.pone.0167356)
Supplement: S5 Table — MIC values for VSSA and hVISA strains determined by CellDirector 3D, Etest and Macrodilution from pure cultures and from spiked blood culture bottles. (PDF) [file pone.0167356.s005.pdf]

S5 Table. MIC values from CellDirector 3D, Etest and macrodilution for VSSA and hVISA.

MIC values for VSSA and hVISA strains determined by CellDirector 3D, Etest and Macrodilution from pure cultures and from spiked blood culture bottles.

	Replicate #	Etest	Macrodilution	CellDirector 3D	
				without blood	with blood
VSSA	1	1.5	0.5	1.3	1.4
	2	1.5	1	0.86	0.93
	3	1.5	1	1.1	1.2
hVISA	1	2	1	2.9	2.5
	2	2	2	2.5	2.3
	3	2	2	1.9	1.7
