# Supplementary material for: A Novel Microfluidic Assay for Rapid Phenotypic Antibiotic Susceptibility Testing of Bacteria Detected in Clinical Blood Cultures
Source: PLoS One. 2016 Dec 14;11(12):e0167356. doi: 10.1371/journal.pone.0167356 (PMC5156554; doi:10.1371/journal.pone.0167356)
Supplement: S6 Table — Percent recovery and bacterial concentrations before and after centrifugation of spiked blood bottles. (PDF) [file pone.0167356.s006.pdf]

S6 Table. Recovery of bacteria from spiked blood bottles. Percent recovery and bacterial concentrations before and after centrifugation of spiked blood bottles.

	Before (CFU/mL)	After (CFU/mL)	Recovery (%)
	$4.4 \cdot 10^{6}$	$2.4 \cdot 10^{6}$	56
VSSA	$4.3 \cdot 10^{6}$	$1.3 \cdot 10^{6}$	30
	$2.9 \cdot 10^{6}$	$2.3 \cdot 10^{6}$	78
	$1.0 \cdot 10^{7}$	$2.4 \cdot 10^{6}$	24
hVISA	$8.6 \cdot 10^{6}$	$1.8 \cdot 10^{6}$	21
	$7.3 \cdot 10^{6}$	$3.6 \cdot 10^{6}$	49
Mean (SD)			43 (20)
