# Supplementary material for: A Novel Microfluidic Assay for Rapid Phenotypic Antibiotic Susceptibility Testing of Bacteria Detected in Clinical Blood Cultures
Source: PLoS One. 2016 Dec 14;11(12):e0167356. doi: 10.1371/journal.pone.0167356 (PMC5156554; doi:10.1371/journal.pone.0167356)
Supplement: S7 Table — Percent recovery and bacterial concentrations before and after centrifugation of clinical blood bottles. (PDF) [file pone.0167356.s007.pdf]

S7 Table. Recovery of bacteria from clinical blood bottles. Percent recovery and bacterial concentrations before and after centrifugation of clinical blood bottles.

Isolate Nr.	Before (CFU/mL)	After (CFU/mL)	Recovery (%)
1	$9.4 \cdot 10^{8}$	$9.7 \cdot 10^{7}$	10
2	$1.1 \cdot 10^{8}$	$4.5 \cdot 10^{7}$	41
3	$1.2 \cdot 10^{9}$	$1.7 \cdot 10^{8}$	14
4	$1.7 \cdot 10^{9}$	$1.5 \cdot 10^{7}$	1
5	$1.9 \cdot 10^{8}$	$8.1 \cdot 10^{6}$	4
6	$5.9 \cdot 10^{7}$	$4.6 \cdot 10^{6}$	8
7	$8.6 \cdot 10^{7}$	$8.9 \cdot 10^{7}$	104
8	$5.3 \cdot 10^{8}$	$6.5 \cdot 10^{7}$	12
9	$3.6 \cdot 10^{7}$	$1.5 \cdot 10^{7}$	41
10	$2.7 \cdot 10^{7}$	$2.8 \cdot 10^{6}$	10
11	$1.9 \cdot 10^{8}$	$1.8 \cdot 10^{7}$	9
12	$5.2 \cdot 10^{7}$	$5.1 \cdot 10^{7}$	96
13	$1.6 \cdot 10^{8}$	$1.2 \cdot 10^{7}$	8
Mean (SD)			28 (33)
