# Supplementary material for: A Novel Microfluidic Assay for Rapid Phenotypic Antibiotic Susceptibility Testing of Bacteria Detected in Clinical Blood Cultures
Source: PLoS One. 2016 Dec 14;11(12):e0167356. doi: 10.1371/journal.pone.0167356 (PMC5156554; doi:10.1371/journal.pone.0167356)

S1 Figure. Population analysis of VSSA and hVISA with vancomycin.

Population analysis of VSSA and hVISA strains using vancomycin agar plates with 0, 0.25, 0.5, 1, 2, 3, 4 and 5 mg/L antibiotic concentration and overnight incubation. The number of surviving colonies represent fraction of resistant sub-populations at the tested concentration. Error bars denote standard deviation (n=2).

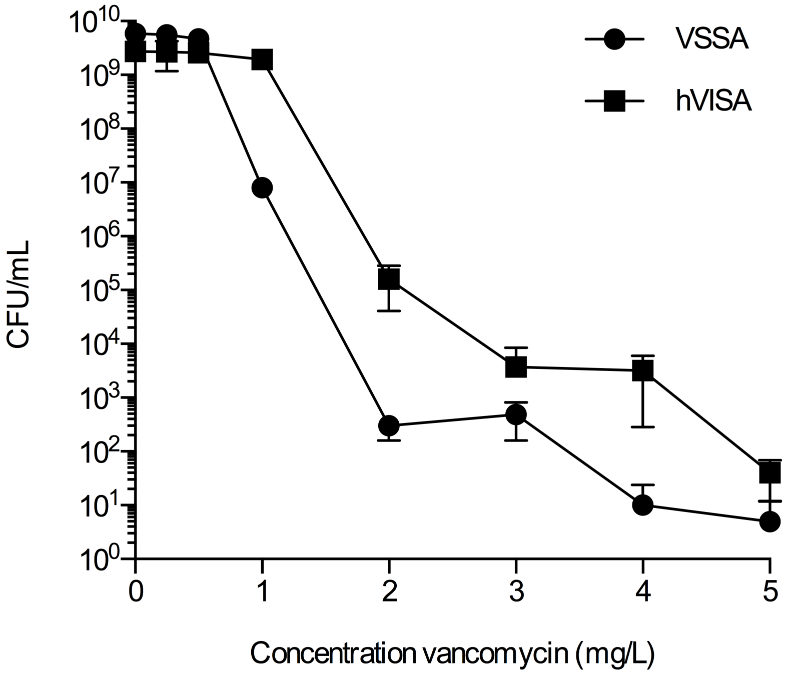

Supplement: S1 Fig — Population analysis of VSSA and hVISA strains using vancomycin agar plates with 0, 0.25, 0.5, 1, 2, 3, 4 and 5 mg/L antibiotic concentration and overnight incubation. The number of surviving colonies represent fraction of resistant sub-populations at the tested concentration. Error bars denote standard deviation (n = 2). (PDF) [file pone.0167356.s008.pdf]
