# Supplementary material for: A Novel Microfluidic Assay for Rapid Phenotypic Antibiotic Susceptibility Testing of Bacteria Detected in Clinical Blood Cultures
Source: PLoS One. 2016 Dec 14;11(12):e0167356. doi: 10.1371/journal.pone.0167356 (PMC5156554; doi:10.1371/journal.pone.0167356)

S2 Figure. Time to readout in CellDirector 3D for clinical blood cultures.

Thirteen isolates of *S. aureus* were extracted from blood bottles after positive signal for growth in BacT/Alert® 3D and identification using microscopy and coagulase test. The samples were analysed in the CellDirector 3D system by collecting an image every 10 minutes. The calculated MIC values are plotted for each time point.

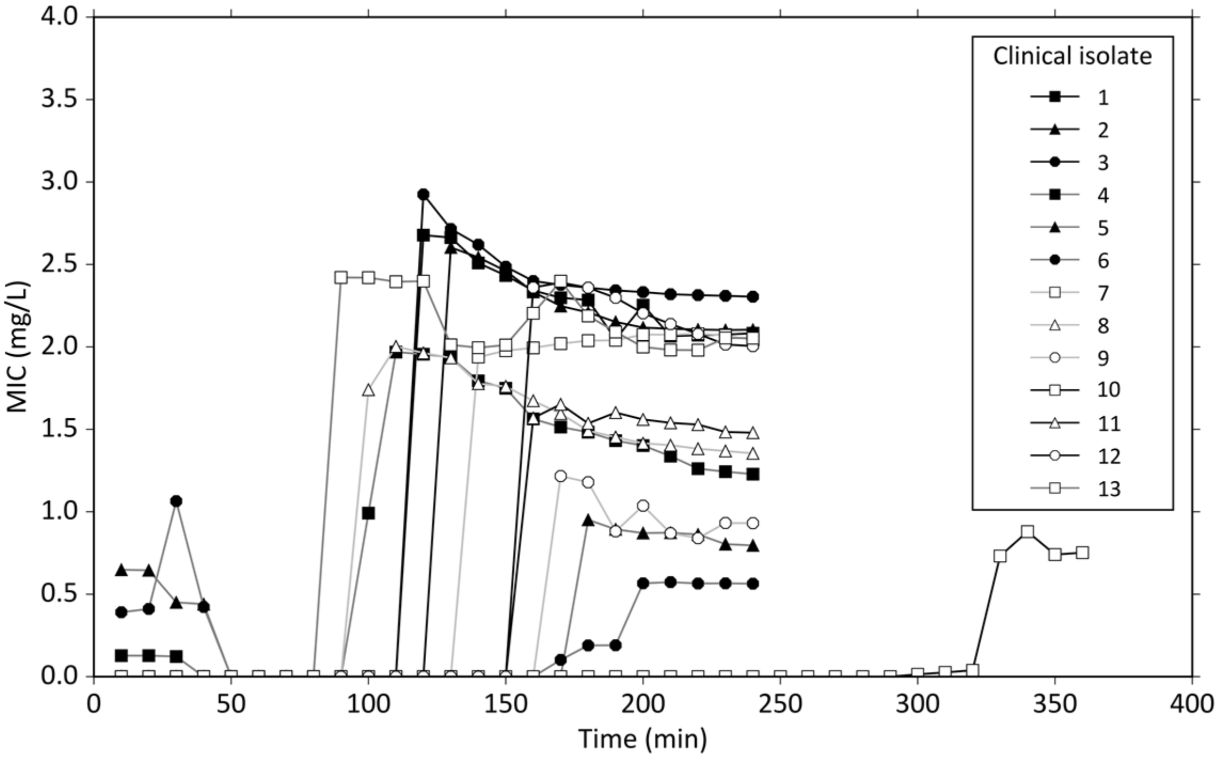

Supplement: S2 Fig — Thirteen isolates of S. aureus were extracted from blood bottles after positive signal for growth in BacT/Alert® 3D and identification using microscopy and coagulase test. The samples were analysed in the CellDirector 3D system by collecting an image every 10 minutes. The calculated MIC values are plotted for each time point. (PDF) [file pone.0167356.s009.pdf]
