# Supplementary material for: A Novel Microfluidic Assay for Rapid Phenotypic Antibiotic Susceptibility Testing of Bacteria Detected in Clinical Blood Cultures
Source: PLoS One. 2016 Dec 14;11(12):e0167356. doi: 10.1371/journal.pone.0167356 (PMC5156554; doi:10.1371/journal.pone.0167356)
Supplement: S1 Text — (PDF) [file pone.0167356.s010.pdf]

S1 Text. Diffusion of vancomycin in CellDirector3D.

One major constraint with the CellDirector 3D method is that in order to have the width of the chamber to represent different antibiotic concentrations a steady linear gradient must have been formed before MIC can be determined. The CellDirector 3D system can be described as a volume bounded by two reservoirs, where diffusion into the system occurs (S1 Figure 1).

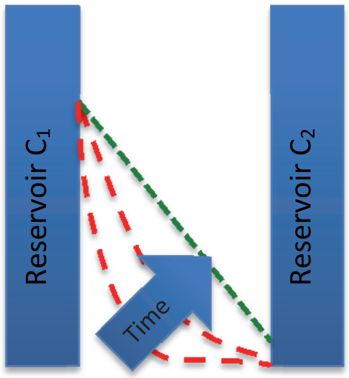

S1 Figure 1. Concentration profile of the diffusing substance in the porous media volume as function of time.

If we disregard the walls, i.e. special boundary conditions, this can be simplified to a problem of one-dimensional diffusion between the planes, i.e. a thin membrane.

Until the concentration distribution reaches a steady state, the concentrations change according to the trigonometrical series presented below (equation 1) where C_0 is the membranes initial uniform concentration (=0), C_1 constant concentration (start. conc.) at x=0, and C_2 constant concentration (=0) at x=l. For large t the terms involving the exponentials vanish and a linear distribution is obtained.

$$C = C_1 + (C_2 - C_1)\frac{x}{l} + \frac{2}{\pi}\sum_{n=1}^{\infty}\frac{C_2\cos(n\pi - C_1)}{n}\sin\left(\frac{n\pi x}{l}\right)\exp(-Dn^2\pi^2 t/l^2)$$

$$+\frac{4C_0}{\pi}\sum_{m=0}^{\infty}\frac{1}{2m+1}\sin\left(\frac{(2m+1)\pi x}{l}\right)exp\left\{-\frac{D(2m+1)^2\pi^2 t}{l^2}\right\} \quad \mathbf{(1)}$$

Methods for reaching this solution are further elaborated in The Mathematic of Diffusions (Crank 1975), and follow from either separation of the variables or by Laplace transform.

The equation above (equation 1) is used to calculate the time until a steady gradient is reached but first, the diffusion coefficient, D, for vancomycin in 0.25% agarose must be estimated. The diffusion coefficient for vancomycin in 1.7% agar is 0.72 (Jones 2006). Furthermore, the diffusion coefficient is assumed to increase as a power law function of volumetric moisture content, i.e. the agar concentration (Mujumdar 2006). To use this relationship we need empirical data on the compound specific behaviour. An idea of this can be obtained from the behaviour of the large molecular species methylene blue, which has been extensively characterized with regard to agar concentration (Vilca-Quispe *et al*. 2010). Fitting a power law relationship to this data and extrapolating to the known vancomycin behaviour at 1.7% agarose to 0.25% agarose yields a diffusion coefficient of approximately 2.7. This diffusion coefficient leads to a fully steady gradient within 2-3 hours (S1 Figure 2).

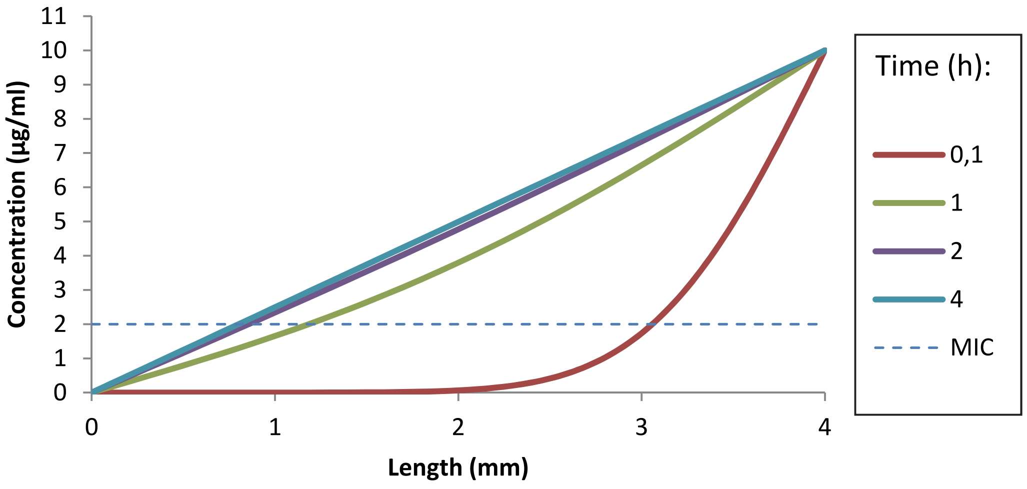

S1 Figure 2. *A linear gradient is reached between 2-3 hours according to equation 1 with a diffusion coefficient of 2.7.*

One can also look at the experimental data and try to estimate the time until a linear steady gradient has been established by determining the time point where the growth rate at the MIC concentration point reaches zero. Here we see that we have a complete linear gradient formation after approx. 180 min (S1 Figure 3).

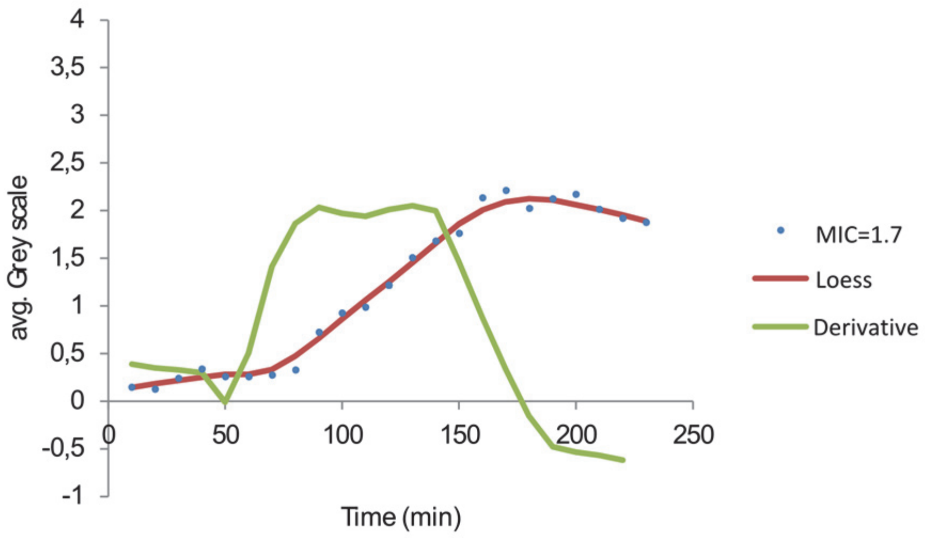

S1 Figure 3. Growth at the MIC concentration point, S. aureus (VSSA) and vancomycin. *The Growth at the MIC is plotted against time. When the derivative (growth rate) is 0 at this point, a linear gradient has been established.*
